# From Poverty Alleviation to Well-Being Enhancement: Empowering Mid-level Managers of JEEViKA and Its Technical Support Program (JTSP) Staff for Health, Nutrition and Sanitation Integration

**DOI:** 10.7759/cureus.70783

**Published:** 2024-10-03

**Authors:** Pragya Kumar, Shamshad Ahmad, Ria Roy, Rakesh K Jha, Swati Swati, Sudarshan Das, Apolenarius Purty, Somya Somya, Narottam Pradhan

**Affiliations:** 1 Community and Family Medicine, All India Institute of Medical Sciences, Patna, Patna, IND; 2 Community Medicine, Santiniketan Medical College, Bolpur, IND; 3 Health and Nutrition, Project Concern International, Patna, IND; 4 Capacity Building and Communications, Project Concern International, Patna, IND; 5 Women Economic Advancement, Project Concern International, Patna, IND; 6 Health and Nutrition, Bihar Rural Livelihoods Promotion Society JEEViKA, Patna, IND; 7 Family Health and Nutrition, Project Concern International, Patna, IND

**Keywords:** classroom training, e-learning modules, health integration, jeevika, jtsp, lms, mid-level managers, rural livelihoods, training need assessment

## Abstract

Introduction: The JEEViKA program, a rural livelihood initiative, sought to uplift Self-Help Group (SHG) cadres in Bihar, India. However, health-related issues remained a challenge due to limited attention and health literacy among SHG members and mid-level managers. This study aimed to enhance health knowledge among mid-level managers through a technical course developed by the All India Institute of Medical Sciences (AIIMS) Patna in collaboration with JEEViKA and Project Concern International (PCI), focusing on integrating health aspects with economic development.

Methods: A comprehensive training needs assessment identified gaps in health knowledge among mid-level managers. A technical course curriculum was developed, comprising 20 topics covering health, nutrition, and sanitation. The course was delivered virtually to six batches of mid-level managers. Pre- and post-training assessments measured knowledge improvement.

Results: Initial assessment revealed that 71 (38.8%) participants had good, while 90 (49.2%) had average health knowledge scores. The virtual course led to improved knowledge levels, with 538 (89.7%) out of 600 total participants achieving good or excellent scores in post-training assessments. Specific knowledge gaps related to maternal, infant, and child health were addressed throughout the course.

Discussion: The study underscores the importance of equipping mid-level managers with health literacy to effectively integrate health components into livelihood projects. The collaboration between AIIMS Patna, JEEViKA, and PCI highlights the potential of knowledge-based interventions to bridge health gaps in rural communities. The success of the virtual course emphasizes the feasibility of online training to enhance health knowledge and underscores the symbiotic relationship between health and economic development.

## Introduction

The Government of Bihar initiated the JEEViKA program under the State Rural Livelihoods Mission to empower rural women through Self-Help Group (SHG) cadres [1]. While the program aimed at fostering economic growth, it faced challenges in addressing health-related concerns within rural communities [2]. Crucially, health issues often received limited attention from both SHG members and Mid-Level Managers responsible for program coordination. This emphasis on economic aspects occasionally overshadowed the equally vital health dimensions, resulting in inadequate health literacy and suboptimal health outcomes [3-5]. Recognizing the need for a comprehensive approach, JEEViKA introduced courses, with technical assistance from Project Concern International (PCI), on Health and Nutrition Interventions (HNS) to bridge the health gap to address the health and nutrition concerns prevalent in these communities. However, it became evident that mid-level managers needed enhanced health literacy to seamlessly integrate these interventions into livelihood projects. In response, AIIMS Patna, in collaboration with JEEViKA and PCI, designed and implemented a tailored technical course for mid-level managers. This course aimed to elevate health awareness, underlining its symbiotic relationship with economic development, thereby contributing to the holistic upliftment of communities. The objectives of this course were multifaceted and included enhancing health literacy among mid-level managers, enabling them to effectively integrate health components into livelihood initiatives, and thereby mitigating the health-related challenges faced by rural communities.

## Materials and methods

Study setting and design

The methodology adopted for the collaborative initiative involved a systematic approach to developing and implementing a technical course on health, nutrition, and sanitation for Mid-Level Managers. The process encompassed several key milestones, each contributing to the successful integration of health into the realm of rural livelihoods.

A Memorandum of Understanding (MoU) was formed between Project Concern International (PCI) and All India Institute of Medical Sciences (AIIMS) Patna on 15th January 2020 to conduct a Training Needs Assessment and then followed up with the conduction of a hybrid method of training with the help of LMS (Learning Management System). The Training Needs Assessment was conducted as a cross-sectional study. All the trainings were conducted at AIIMS Patna either offline or through online LMS modules shared with the participants.

Sample size

A rapid survey was conducted to evaluate the baseline knowledge of the participants, which comprised District Program Managers, Managers of Nutrition of JEEViKA, and JEEViKA Technical Support Program (JTSP) staff, regarding fundamental health and nutrition components. The survey was administered through an online Google Form, and 183 responses were obtained, which consisted of Block Project Managers (162), District Project Managers (5), Health and Nutrition Managers (14), Manager-Human Resources (1), and Manager-Bank Linkage and Micro-finance (1). These 183 respondents represented proportionally all the mid-level managers of JEEViKA in the state.

Study procedure

There were four milestones in the course of completion of training.

Milestone 1: Training Needs Assessment (TNA)

A critical starting point was conducting a Training Need Assessment (TNA) to understand the knowledge gaps and learning requirements of the target audience, which comprised District Program Managers, Managers of Health and Nutrition of JEEViKA, and JTSP staff. The assessment on 31st January 2020 involved interactions with key stakeholders and a cross-sectional study to evaluate the baseline knowledge of the participants regarding health, nutrition, and sanitation components.

The participants' overall knowledge was subsequently assessed utilizing a grading system defined by percentile scores. Participants achieving scores above 70% were classified as having "Excellent" knowledge, those within the range of 50-70% were categorized as "Good," and those below 50% fell into the "Average" knowledge group. This survey served as a crucial precursor, enabling a clearer understanding of the participants' knowledge gaps and shaping subsequent training initiatives.

Milestone 2: Imparting Classroom Training at AIIMS Patna

It encompassed the implementation of classroom training at AIIMS Patna in April 2020, based on the insights and knowledge gap learned from the Training Needs Assessment in Milestone 1. A comprehensive technical module focusing on the integration of health and nutrition within the Rural Livelihoods Mission was meticulously designed by the AIIMS Patna team. These modules were tailored to accommodate both in-person classroom training and self-paced e-learning modules, ensuring flexibility for diverse learning preferences. The course comprised 20 discrete topics, including key themes such as the interplay between health, nutrition, and livelihoods, the critical significance of the first 1000 days in shaping children's future, maternal and child healthcare, and the dynamics of disease identification and management.

Initially planned as offline training sessions conducted over three days each, the outbreak of the COVID-19 pandemic necessitated an adaptation to virtual training mode using the Zoom platform. The virtual classroom sessions were conducted for approximately 2.5 to 3 hours each day over six days for each batch, allowing for in-depth exploration of each topic. Participants were presented with a range of health-related questions through a Google Form, originally structured as a quiz format to assess their comprehension. However, in response to feedback and to eliminate potential confusion, the form was converted to a survey mode after the initial responses. Feedback was systematically collected from participants and analyzed, with adjustments made to the course content and delivery approach as warranted. The process was iterative, with successive batches receiving enhancements based on previous participants' input. The virtual format allowed for broader participation, enabling timely completion of the training despite the pandemic's challenges.

Milestone 3: Designing and Developing e-Learning Contents

Simultaneously, while the classroom training was ongoing, the AIIMS Patna team started developing interactive e-learning modules called LMS (Learning Management System). These modules were designed to complement the classroom sessions and provide a flexible and self-paced learning experience. Each e-learning module consisted of short virtual sessions (12-15 minutes each) on specific topics related to health, nutrition, and sanitation which were created by the relevant subject experts associated with the training program. The modules were designed to cater to diverse learning preferences and were created in both English and Hindi to ensure accessibility. The modules were completed by 15th December 2020. To date, they have been made accessible to around 600 block project managers, health and nutrition managers, and other cadres under JEEViKA.

Milestone 4: Preparation of Final Exam Questions and Certification

After completing the classroom training and e-learning modules, all participants of the modules were assessed through a final examination on 15th March 2021. The examination, conducted online via LMS modules, consisted of 50 objective-based questions related to the topics covered in the course. Participants needed to achieve a score of at least 50% to pass the assessment. The results were automatically calculated, and participants who completed the assessment were awarded certificates to recognize their achievement in integrating health into rural livelihoods.

Ethical approval

All procedures performed in this study were by the ethical standards of the institutional research committee (IRC) and with the 1964 Helsinki Declaration and its later amendments or comparable ethical standards. The study was approved by Project Concern International and the Institutional Research Committee of AIIMS Patna (approval number: PCI-1411-SC-0610-00). Informed consent was obtained from all individual participants included in the study.

## Results

Milestone 1: training needs assessment (TNA)

Table 1 presents a comprehensive overview of the demographic characteristics and the corresponding knowledge scores of the initial 183 participants for the training needs assessment. In terms of age distribution, 103 (56.4%) participants fell within the age bracket of 31 to 40 years. There were 38 (20.7%) participants, aged 20 to 30 years and 41 to 50 years old. This distribution underscores the engagement of a diverse age group, contributing to a richer exchange of experiences and perspectives. Regarding gender composition, the table shows that a significant majority of participants, that is 156, were male (85.3%).

**Table 1:** Distribution of the participants according to their demographic characteristics and overall knowledge (n=183)

Characteristics	Frequency	%	95% CI
Age (in years)			
20-30	38	20.7	14.9-26.6
31-40	103	56.4	49.1-63.5
41-50	38	20.7	14.9-26.6
>50	4	2.2	0.1-4.3
Gender			
Male	156	85.3	80.1-90.4
Female	26	14.2	9.2-19.3
Not mentioned	1	0.5	0.0-1.6
Overall Knowledge score			
Excellent (>70%)	22	12	7.3-16.7
Good (50-70%)	71	38.8	31.7-45.8
Average (<50%)	90	49.2	41.9-56.4

The most vital facet of this table is the participants' overall knowledge scores, which were determined based on their performance in the pre-training assessment. A larger portion, 71 (38.8%), achieved a "Good" knowledge level, signifying a strong foundational grasp of health and nutrition concepts. Notably, 90 (49.2%) fell into the "Average" knowledge category, emphasizing the opportunity for targeted training interventions to elevate their understanding.

Furthermore, the survey delved into specific areas of health and nutrition, divulging insights into the participants' understanding of key concepts. Notable findings emerged, including a comprehensive understanding of antenatal care and birth preparedness, with an overwhelming majority correctly identifying crucial components. Responses also highlighted areas where misconceptions prevailed, such as the calculation of measles vaccination dates based on the child's birth date. The survey outcomes laid the foundation for tailoring subsequent training modules to address the identified gaps and to fortify the participants' grasp of essential health and nutrition principles.

Table 2 presents the distribution of respondents’ specific knowledge related to maternal, infant, and child health across various domains. In the realm of Obstetric and Antenatal Care Knowledge (Section A), the survey reflected that 91 (49.7%) participants were aware of the standard five-day duration of menstruation, while 150 (82.0%) recognized urine tests as a method for pregnancy testing at Government health centers. The survey also revealed that 122 (66.7%) acknowledged the importance of a minimum of four antenatal visits. In the context of the Management of Diarrhoea (Section B), 137 (74.8%) participants recognized the use of Oral Rehydration Solution (ORS) as an effective community-level intervention, whereas only two (1.1%) considered hospital treatment. Moreover, the optimal time window for consuming ORS within 24 hours was identified by 110 (60.1%) participants. The section on Nutrition of Infants and Children (Section C) unveiled that 78 (43.3%) were aware of the proportion of infants in Bihar exclusively breastfed, and an overwhelming 177 (97.3%) highlighted a lack of knowledge about child nutrition as a significant cause of malnutrition among under-five children. Within Immunization of Infants and Children (Section D), 153 (84.5%) recognized Tetanus as an important vaccine for both pregnancy and newborns, while 125 (68.8%) identified the Pentavalent vaccine as protective against five diseases. Finally, the section on Nutrition of Mothers (Section E) illuminated that 92 (50.5%) were aware that a weight gain of 10-12 kg is recommended during pregnancy and 114 (63.4%) recognized the importance of consuming an extra meal. This comprehensive assessment offers valuable insights into the current state of health-related knowledge among the surveyed participants, highlighting both areas of strength and potential for improvement in maternal, infant, and child health practices.

**Table 2:** Distribution of respondents according to specific knowledge of the maternal, infant, and child health Govt.: Government, LMP: Last menstrual period, ASHA: Accredited Social Health Activists, ORS: Oral rehydration solution, BCG: Bacillus Calmette–Guérin.

A. Obstetric and antenatal care knowledge	Frequency (%)	95% CI
The average duration of menstruation (in days) (n=183)		
Three days	64 (35.0)	28.1-41.2
Five days	91 (49.7)	42.5-56.9
Six days	3 (1.6)	0.0-3.5
Seven days	25 (13.7)	8.7-18.6
Pregnancy testing available at Govt. health center (n=183)		
Blood test	5 (2.7)	0.4-5.1
Urine test	150 (82.0)	76.4-87.5
Ultrasound	6 (3.3)	0.7-5.8
From LMP	22 (12.0)	7.3-16.7
Minimum number of antenatal visits (n=183)		
One	1 (0.5)	0.0-1.6
Two	7 (3.8)	1.1-6.6
Three	49 (26.8)	20.4-33.2
Four	122 (66.7)	59.8-73.5
No answer	4 (2.2)	0.1-4.3
Birth preparedness steps include (n=183; Multiple responses permitted)		
Identification of hospital	173 (94.5)	90.5-97.2
Number of ambulance/vehicles	155 (84.7)	78.9-89.4
Number of ASHA	160 (87.4)	87.4-91.7
Financial preparation	138 (75.4)	75.4-81.2
Identification of blood donor	145 (79.2)	79.2-84.7
B. Management of diarrhoea		
The most effective method for the management of diarrhoea at the community level (n=183)		
Hospital treatment	2 (1.1)	0.0-2.6
Use of ORS	137 (74.8)	68.6-81.2
Use of toilets	31 (17.0)	11.5-22.4
Handwashing with soap	13 (7.1)	3.4-10.8
The time duration within which the ORS preparation should be consumed (n=183)		
1 hour	44 (24.0)	17.8-30.2
6 hours	10 (5.5)	2.2-8.7
12 hours	19 (10.4)	5.9-14.8
24 hours	110 (60.1)	53.0-67.2
C. Nutrition of infants and children		
Number of infants out of 10 who get exclusively breastfed in Bihar (n=180)		
3	52 (28.9)	22.3-35.5
5	78 (43.3)	36.1-50.6
7	38 (21.1)	15.2-27.1
9	12 (6.7)	3.0-10.3
The most important cause of malnutrition among under-five children (n=182)		
Lack of knowledge about the child’s nutrition	177 (97.3)	94.9-99.6
The family below the poverty line	5 (2.7)	0.4-5.1
Lack of facilities	0 (0.0)	0-0
Deficiency of Govt. policies/programs	0 (0.0)	0-0
D. Immunization of infants and children		
Vaccine which is important for both pregnancy and newborn (n=181)		
Tetanus	153 (84.5)	79.3-89.8
Hepatitis B	21 (11.6)	6.9-16.3
BCG	6 (3.3)	0.7-5.9
Measles	1 (0.6)	0.0-1.6
Number of diseases against which the Pentavalent vaccine protects (n=182)		
3	27 (14.8)	9.7-20.0
4	15 (8.2)	4.3-12.2
5	125 (68.8)	61.9-75.4
6	15 (8.2)	4.3-12.2
Timing of measles vaccine (n=143)		
After six months of birth	30 (21.0)	14.3-27.6
After nine months of birth	102 (71.3)	63.9-78.7
After 12 months of birth	11 (7.7)	3.3-12.1
E. Nutrition of mother		
Average weight gain during pregnancy (in kgs) (n=182)		
4-6 Kg	38 (20.9)	14.9-26.7
6-8 Kg	24 (13.2)	8.3-18.1
8-10 Kg	28 (15.4)	10.1-20.6
10-12 Kg	92 (50.5)	43.3-57.8
Frequency of meals during pregnancy (n=180)		
Two times	8 (4.4)	1.4-7.4
Three times	58 (32.2)	25.4-39.1
One extra meal	114 (63.4)	56.3-70.4

Milestone 2: imparting classroom training at AIIMS Patna

Milestone 2 marked the commencement of training delivery, both in online classrooms and through e-learning modules. A total of around 600 participants were enrolled and trained across six batches, encompassing a diverse array of profiles, including Block Project Managers, Health & Nutrition Managers, and other vital roles. The online modules showed a gradual decline in attendance as the training progressed. Notably, participation in modules addressing planned family methods saw a lower engagement rate.

Milestone 3: designing and developing e-learning content

Recognizing the significance of flexibility and scalability, an e-learning module was developed as part of the project's progression. These modules aimed to further broaden the accessibility of the training material, enabling participants to engage with the content at their own pace and convenience. The e-learning module encompassed the same 20 topics covered in the classroom training, ensuring consistency in knowledge dissemination. By adopting a blended approach, combining classroom training with self-paced e-learning, the project extended its impact to a wider audience, accommodating varied learning preferences and constraints.

More than 90% of almost 600 participants completed modules, reflecting the accessibility and effectiveness of the digital learning format. Feedback from participants highlighted the positive impact of the sessions and their appreciation for the incorporation of short videos to enhance understanding.

Milestone 4: preparation of final exam questions and certification

A post-training evaluation and analysis were conducted to gauge the effectiveness of the training interventions. The responses gathered from participants were carefully analyzed, shedding light on both the strengths and areas that required further enhancement. The feedback encompassed diverse aspects such as the clarity of content, the effectiveness of virtual sessions, and the overall learning experience. Valuable insights derived from the feedback played a pivotal role in refining future training modules, adapting the content to align with participants' expectations, and fine-tuning the delivery methods.

It culminated in the final assessment and provided a comprehensive evaluation of participants' knowledge acquisition. The results revealed an average score of 42 ± 9, indicating a promising level of comprehension. Impressively, 538 (89.7%) of participants successfully passed the assessment, showcasing the effectiveness of the training initiative.

## Discussion

In discussing the implications of our findings, it is noteworthy to acknowledge the prevailing trend in the existing literature regarding the role of online training in enhancing knowledge and skills in the realm of health and disease. The majority of available literature in this domain predominantly focuses on individuals within the healthcare system, such as medical professionals, nurses, and allied health workers. While these studies have provided valuable insights into the efficacy of eLearning in addressing the needs of the healthcare workforce, it is essential to recognize that our study's participants, Mid-Level Managers from JEEViKA and JTSP, belong to a distinct category. As non-health cadres, their roles encompass a broader scope, often involving community engagement, livelihood enhancement, and program management at secondary and tertiary administrative levels [6]. By delving into the specific challenges and opportunities faced by Mid-Level Managers in the health, nutrition, and sanitation sectors, our study contributes to the expanding discourse on the role of online training, offering insights that resonate with their distinctive responsibilities and expertise. Implementing e-learning modules in health leadership and management, barriers such as limited time, resources, access to experts, and perceived relevance can be overcome, resulting in enhanced knowledge, skills, attitudes, behaviors, and satisfaction among Mid-Level Managers [7-10]. These e-learning modules can be used in other regions of the country with similar issues among mid-level managers regarding knowledge of Health and Nutrition. This contextualized approach aids in better understanding the potential benefits and constraints of eLearning in non-traditional health roles, guiding future research and policy initiatives in this often overlooked but vital aspect of capacity building.

A couple of observations were: The learning curiosity increased among the mid-level managers when the importance of HNS was linked with their core jobs, which was around livelihood: (1) Designing courses around the association of livelihood is significantly based on health, nutrition, and sanitation; (2) E-learning and automated examination create more space for users to learn by themselves. However, quality module preparation and core contents are important; (3) Continuous mentorship is seen to be important to teaching non-subject managers.

In line with the findings from the systematic review by Tudor Car et al. [11], which explored the role of eLearning in health management and leadership capacity building within health systems, our study also underscores the potential of online training in enhancing the knowledge and skills of health managers. The systematic review highlighted the advantages of eLearning, such as its flexibility, accessibility, and potential to bridge geographical barriers. Similarly, our study demonstrated the efficacy of online training, particularly during the unprecedented challenges posed by events like the COVID-19 pandemic. Both studies converge on the idea that eLearning can play a pivotal role in capacitating health managers by offering tailored courses and addressing specific gaps in knowledge. However, our study goes further by focusing on the unique context of delivering technical courses in Health, Nutrition, and Sanitation to mid-level managers, a population crucial for effective program implementation at the grassroots level. By adapting the principles highlighted in the systematic review, our project effectively designed and delivered course content through virtual platforms, providing valuable insights into the potential benefits and challenges of eLearning specifically in the context of our targeted audience.

Drawing a comparative perspective from the study conducted by Elzainy et al. [12], which explored the experience of e-learning and online assessment during the COVID-19 pandemic in a medical education context, provides valuable insights for our discussion. The College of Medicine, Qassim University, focused on medical education, represents a specific domain where the transition to online learning was necessitated by unprecedented circumstances. This study underscores the significance of adapting pedagogical approaches to virtual platforms and highlights the challenges faced by educators and learners in the process. While our study addresses a distinct demographic, namely mid-level managers from JEEViKA and JTSP, operating in the fields of health, nutrition, and sanitation, parallels can be drawn in terms of the need for tailored online training solutions. Both studies emphasize the need for interactive and engaging online learning experiences, aligned with the participants' roles and responsibilities. The lessons learned from the medical education context provide insights that resonate with our efforts to enhance the capacity of non-health cadres, albeit in a different professional realm. As such, our study contributes to the broader discourse on e-learning by extending its relevance to varied sectors, demonstrating its adaptability to diverse contexts, and aligning with the changing dynamics of education and skill development in the digital age.

During the "Health Assembly, Bihar-2018" organized by Jan Swasthya Abhiyan (JSA), concerns over healthcare accessibility and affordability were brought to the forefront. The assembly shed light on the alarming out-of-pocket healthcare expenditures, particularly noteworthy considering our manuscript's central objective. The assembly emphasized the need to transform healthcare into a fundamental right, given the stark reality that total health expenditure per capita in Bihar is Rs 2047, with out-of-pocket expenditure accounting for a striking 82.3% of this total. This financial strain pushes a significant population toward the below-poverty line category [13].

The study titled "Out-of-pocket expenditure on prenatal and natal care post-Janani Suraksha Yojana: a case from Rajasthan, India," conducted by Govil et al. [14] presents a relevant case from Rajasthan, India, regarding out-of-pocket healthcare costs during prenatal and natal care despite the implementation of the Janani Suraksha Yojana. This study's findings emphasize the persistent financial burden borne by families even after the implementation of government healthcare initiatives. This situation aligns with our context, where we aim to empower mid-level managers in JEEViKA to mitigate out-of-pocket healthcare expenditures among self-help group families. This study underscores the urgency of such interventions and highlights the need for comprehensive strategies to tackle healthcare affordability and accessibility challenges.

The collaborative capacity-building initiative presented in this study signifies a crucial stride toward addressing health literacy gaps and seamlessly integrating health and nutrition interventions within the rural livelihood framework of JEEViKA. The partnership between JEEViKA, PCI, and AIIMS Patna not only exemplifies the importance of community-driven health interventions but also underscores the inherent synergy between health and economic development goals. The outcomes of this initiative offer valuable insights into the potential for transformative impact on both health outcomes and community livelihoods. The shift in focus from economic development alone to a comprehensive approach encompassing health and nutrition interventions is pivotal for realizing sustainable development objectives [15]. The initiative recognized that health and livelihoods are inherently interconnected, and the success of one is contingent upon the well-being of the other. By enhancing the health literacy of mid-level managers, this collaborative effort empowers them to effectively integrate health components into the fabric of livelihood projects. This integration, in turn, holds the promise of improved health outcomes and reduced health-related expenditures within the community.

The incorporation of e-learning modules emerged as a noteworthy component of this initiative. The high completion rate of Module 1 and positive feedback underscored the accessibility and effectiveness of digital learning formats. The scalability and flexibility of e-learning modules address constraints related to time and geographical barriers. This experience highlights the potential of digital solutions to access essential knowledge and training, ultimately contributing to equitable development across diverse settings. The positive outcomes observed in the final assessment underscore the efficacy of this capacity-building initiative. The average score of 42 ± 9 reflects a promising level of comprehension and understanding among participants. This success validates the relevance and appropriateness of the curriculum designed by AIIMS Patna. Moreover, the high pass rate of 89.7% demonstrates the initiative's potential to equip mid-level managers with the requisite knowledge and skills to contribute meaningfully to health and nutrition integration.

Given the limited availability of literature specifically tailored to this unique group, our discussion takes this into account in the context of available evidence. There could have been a self-reporting bias during the Training Needs Assessment as it was conducted online. Another limitation was the lack of field testing of the health delivery component by the mid-level managers instead of only online tests, which could have provided a bigger comprehensive picture of the effectiveness of the e-learning modules.

## Conclusions

The transition from business-focused mindsets to a more comprehensive view that integrates health and nutrition underscores a transformative shift. The mid-level managers, once entrenched in business-oriented priorities, now recognize the significance of health as a cornerstone of holistic development. This shift is crucial for sustainable poverty alleviation, as it acknowledges that robust health, nutrition, and sanitation underpin effective livelihood strategies.

In conclusion, the collaborative efforts of JEEViKA, PCI, and AIIMS Patna exemplify a model that envisions a future where rural development thrives on the integration of health, nutrition, and sanitation. The empowerment of mid-level managers through targeted capacity-building signifies a paradigm shift in rural development strategies, catalyzing sustainable change at both individual and community levels. This initiative holds immense promise not only for Bihar but also as a replicable model for similar endeavors globally.
